# Editorial: Returning to Mechanisms in Psychological Therapies: Understand the Engine Before Steaming in

**DOI:** 10.3389/fpsyt.2021.694088

**Published:** 2021-05-24

**Authors:** Liam Mason, Warren Mansell, David E. J. Linden, Veena Kumari

**Affiliations:** ^1^Max Planck University College London Centre for Computational Psychiatry and Ageing Research, University College London, London, United Kingdom; ^2^Wellcome Centre for Human Neuroimaging, University College London, London, United Kingdom; ^3^Research Department of Clinical, Educational and Health Psychology, University College London, London, United Kingdom; ^4^Division of Psychology and Mental Health, CeNTrUM Centre for New Treatments and Understanding in Mental Health, Manchester Academic Health Science Centre, Faculty of Biology, Medicine and Health, School of Health Sciences, University of Manchester, Manchester, United Kingdom; ^5^Faculty of Health, Medicine and Life Sciences, School for Mental Health and Neuroscience, Maastricht University, Maastricht, Netherlands; ^6^Centre for Cognitive Neuroscience, College of Health, Medicine and Life Sciences, Brunel University London, Uxbridge, United Kingdom

**Keywords:** psychological therapy, psychotherapy, mechanisms, neuroimaging, brain, cbt, cognitive behavioural, computational psychiatry

Many pivotal techniques from evidence-based psychological interventions for other disorders grew from basic research into mechanistic processes. Yet the past few decades have seen the field of psychological interventions drift away from its scientific roots. This year, the Lancet commission for improving psychological treatments (1) urged greater synergy between basic and clinical research, and integration with technology and physiological measures. This special section focuses on bringing a mechanistic focus to psychological therapies and showcases some of the most recent advances in understanding the mechanisms of psychological therapy across ten important theoretical and empirical contributions in the field.

Setting the scene, Carey et al. attempt a review of potential functional mechanisms (i.e., mechanisms that “express plausible actions consistent with known biological processes”) underlying positive outcomes in psychological therapy. Their findings are thought provoking in showing a lack of empirical research on this topic and present a strong case for clinicians and researchers to work together and link psychological intervention practises to scientific theories capable of integrating functional mechanisms and biological processes if we are to advance meaningfully in this area. Next, Watkins and Newbold outline how trial designs can be exploited to better understand how psychological interventions exert their therapeutic effects. Moving beyond the identification of statistical mediation effects, they detail The Multiphase Optimality Strategy; a sophisticated way of assessing the active ingredients of a multi-component psychological intervention by blending different elements within a factorial design.

Going beyond studying the effects of experimental manipulations, Mansell and Huddy build on the view that the most robust test for a psychological mechanism arises from constructing a neurally plausible functional model of the mechanism using a computational framework. They focus specifically on perceptual control theory which provides a mathematical specification of principles that may be fundamental to mental health—namely control, conflict and reorganisation. Their article provides a review of existing studies and describes the methodology of this approach. Nair et al. also advocate a computational approach, and outline how computational psychiatry models rooted within a reinforcement learning framework could be clinically applied to understanding mechanisms in mood disorders, and to make existing therapies more mechanism-focused. They focus on making expectation and reward prediction errors more explicit in behavioural activation and behavioural experiments, and discuss parameters from computational models as useful “read-outs” for evaluating progress in psychological therapy.

Shifting gears to empirical investigation of mechanisms, Di Simplicio et al. examine mental imagery as a potential endophenotype for affective disorder; that is, whether imagery differences are a manifestation of genetic contributions that precede diagnosis. They tested whether imagery differences are present in a group at elevated genetic risk for affective disorders, as well as in a group who had been diagnosed with an affective disorder but were out of episode. They did not find support for an endophenotype account; imagery abnormalities were only present in those who had been diagnosed with an affective disorder, suggesting that they are markers of established affective disorder. The authors argue that this mechanism likely increases vulnerability to future relapse and therefore represents an important target for personalised imagery-based intervention.

Moving from behavioural measures to physiological measures, Skottnik and Linden outline how advances in real-time neurofeedback from functional MRI can be used to advance our understanding of the neural mechanisms of imagery interventions and potentially increase their effectiveness. Like Di Simplicio et al. (2), they stress the role of mental imagery in current psychotherapeutic approaches, particularly in reducing negative biases in depression and for imaginal exposure in anxiety disorders. They detail how insights into the neural mechanisms of mental imagery can give rise to new neuromodulation approaches like neurofeedback, a self-regulation training of brain activity. They argue that a systematic combination with psychotherapeutic techniques will be crucial for the translation of neurofeedback into a clinical tool.

Riedl et al. (3) also harness neurobiological measurements, using functional MRI to investigate the therapeutic mechanism of activation of a novel training intervention for psychosis. Their trial has a particularly innovative combination of outcome measures, covering cognitive performance (e.g., working memory), social skills (e.g., self- and informant assessed communicative abilities) and neural measures (fMRI acquired during the presentation of audiovisual communication signals). It is thus paradigmatic for the topic of this volume, with its combination of clinical and mechanistic evaluation of cognitive-behavioural interventions.

Griffith and Saunders outline the utility of smartphone-based assessments as an objective measure of mood. This technology provides one excellent avenue for testing the computational approaches advocated within this special issue. The article provides an up-to-date overview of this burgeoning area of research, explaining the advantages of this technology for frequent, ecologically valid assessment of data, some of which can be recorded continuously with no effort from the participants. They also clarify the challenges and controversies of this methodology, including issues regarding data security and participant preoccupation with the monitoring technology. A potential direction for physiological and digital assessment is its application to chronic physical illnesses; the first step being to identify the underlying mechanism involved in distress regarding the illness. Khatibi et al. report a psychometric evaluation of a Fear of Relapse scale in patients with Multiple Sclerosis, finding evidence for its distinctiveness, reliability and validity.

Finally, moving to a real example of treatment innovation Hirsch et al. describe how theory-driven empirical research can be used to make cognitive-behavioural interventions for generalised anxiety disorder more mechanism-focused. They isolate worry as an overarching process at the core, and detail a modified 12-session intervention; noteworthy as this is briefer and therefore potentially more efficient than standard interventions. They indeed report reductions in worry and general anxiety with large effects (d = 0.90–2.54) that exceed standard interventions, with the next step being to quantify how much of the added effectiveness is due to this modified approach.

Taken together, the articles in this collection crystallise a scientific approach that advocates precise specification, modelling and measurement of putative mechanisms of psychological therapies, that directly translates to the development of advanced, efficient and targeted interventions. The role of the human therapist is likely to become increasingly scrutinised as these developments in therapy unfold. Are they an expert who applies a well-honed technique to target the mechanism within a compliant client—or a facilitator who uses their understanding of mechanism to flexibly enable active, client-led change?

## Author Contributions

All authors listed have made a substantial, direct and intellectual contribution to the work, and approved it for publication.

## Conflict of Interest

The authors declare that the research was conducted in the absence of any commercial or financial relationships that could be construed as a potential conflict of interest.
